# Evaluation of ENG/CD105 expression, methylation, immuno-response, and cordycepin (CD) regulation as a novel biomarker of breast invasive carcinoma (BRCA)

**DOI:** 10.7150/jca.98767

**Published:** 2024-08-13

**Authors:** Dabing Li, Meiling Zheng, Wenqian Zhang, Jiayue He, Lianmei Zhang, Qi Tan, Mazaher Maghsoudloo, Kemeng Liu, Ting Li, Ranbin Yao, Chunli Wei, Jingliang Cheng, Junjiang Fu

**Affiliations:** 1 Key Laboratory of Epigenetics and Oncology, the Research Center for Preclinical Medicine, Southwest Medical University, Luzhou 646000, Sichuan Province, China.; 2 Department of Rehabilitation Medicine, the Affiliated Hospital of Southwest Medical University, Luzhou 646000, Sichuan Province, China.; 3 School of Basic Medical Sciences, Southwest Medical University, Luzhou 646000, Sichuan Province, China.; 4 Department of Pathology, The Affiliated Huai'an No. 1 People's Hospital of Nanjing Medical University, Huai'an 223300, Jiangsu Province, China.; 5 Department of Infection Management, the Affiliated Traditional Chinese Medicine Hospital, Southwest Medical University, Luzhou 646000, Sichuan Province, China.; 6 Children's Nutrition Research Center, Department of Pediatrics, Baylor College of Medicine, Houston, Texas 77030, United States.

**Keywords:** Breast invasive carcinoma (BRCA), ENG/CD105, Methylation, Diagnostics, Prognostics, Cordycepin (CD)

## Abstract

ENG/CD105 encodes a vascular endothelial glycoprotein and plays a crucial role in modulating angiogenesis. However, the significance of ENG expression, DNA methylation, immuno-response, and cordycepin (CD) regulation as diagnostic, prognostic, and therapeutic markers for breast invasive carcinoma (BRCA) remains unclear. As a result, ENG is decreased in BRCA tissues compared with corresponding healthy tissues. Five isoforms were found, and the utilization for *ENG* isoform (ENG-002) was the highest, suggesting its potential involvement in important roles in BRCA. *ENG* DNA was frequently altered in most types of cancer, and overall survival (OS) for mutant *ENG* was significantly longer than for wild-type cases. High expressions of *ENG* remarkably correlate with long relapse-free survival (RFS) for breast cancer (BC). Additionally, the *ENG* methylation level was higher in BRCA tissues compared with matched healthy tissues. The *ENG* expression and DNA methylation showed a significantly reverse correlation, demonstrating that *ENG* methylation may be a regulatory mechanism. By constructing diagnostic and prognostic models of *ENG* methylation for BRCA, we found four CpGs (CpG sites) that ranked with high importance. High methylation for cg14185922 of ENG in BRCA tissues showed shorter OS (high risk), indicating that *ENG* CpGs' methylation has potential as a diagnostic and prognostic biomarker for BRCA. Moreover, ENG might be a novel target for tumor immune response and immunotherapy in pancancer, including BC. CD, an adenosine analog and anti-cancer agent, increased ENG levels in a dose-dependent manner in animal models. This suggests that CD repressed BC growth and metastasis, at least partially through increasing the expression of the tumor suppressor gene ENG. Thus, our study successfully evaluated ENG/CD105 expression, DNA methylation, immune response, and CD regulation, which act as a novel diagnostic, prognostic, and therapeutic biomarker for BRCA. This research also fills critical knowledge gaps in this ENG/cancer field and highlights ENG's potential importance for the diagnosis, prognosis, and treatment of BRCA.

## Introduction

The *endoglin* (*ENG*) gene (OMIM: 131195), also known as CD105, CD105 antigen, Osler-Rendu-Weber syndrome 1 (ORW1), or Osler-Rendu-Weber syndrome (ORW), encodes a vascular endothelium glycoprotein at 9q34.11. As a homodimeric transmembrane protein, it plays a critical role in the regulation of angiogenesis. ENG is connected with the vascular endothelium and is present on bone marrow proerythroblasts, triggering monocytes and lymphoblasts in childhood leukemia. The ENG complexes with the receptor of transforming growth factor beta (TGFβ), including β1/3, with high affinities 1. The level of vascular endothelial growth factor (VEGF) correlates with TGF-β. NRP1, a SARS-CoV-2 receptor 2, interacts with ENG and VEGFR2 to modulate the VEGF pathway and endothelial cell sprouting 3. Additionally, ENG is affected by the BMP9-regulated signaling 4, 5. ENG mutations cause hereditary hemorrhagic telangiectasia Type 1 (HHT1), a rare autosomal dominant inherited vascular disease, leading to telangiectases and arteriovenous malformations of mucosa, skin, and viscera including the brain, lung, and liver (OMIM: 187300) 6.

The *ENG* gene plays critical roles in cancers. ENG expression has been linked to squamous cell carcinoma (SCC), including head and neck SCC (HNSCC), esophageal SCC (ESCC), and vulvar SCC (VSCC) cancers 7, 8. ENG also plays roles in the progression and therapy of renal cell carcinoma (RCC) 9. ENG expression (but not that of E-cadherin, an EMT marker of cancer) has been reported to be connected with laryngeal cancer recurrence and disease-free interval 10. In tumor-derived mesenchymal progenitor cells (MPCs), stem cell characteristics including CD73, CD90 and CD105, are expressed, and regulated breast cancer (BC) proliferation 11. TRC105 (carotuximab) is a chimeric anti-ENG antibody, while bevacizumab is a VEGF inhibitor. In a pre-registered and multicenter phase II trial study, Ahluwalia MS *et al.* reported that the combination of TRC105 with bevacizumab could have therapeutic efficacy for bevacizumab-refractory glioblastoma (GBM) 12. ENG thus may be a prognostic and therapeutic biomarker in some types of cancer, such as colorectal cancer, acute myeloid leukemia, glioblastoma multiforme (GBM), etc. 13-16. In addition, in red blood cells in bone marrow samples of low-grade myelodysplastic neoplasms (MDS), ENG (+/-) with side scatter (SSC) parameters can be used for diagnosis through flow cytometry 17, 18. However, it is unclear whether ENG plays roles as a biomarker in BC including breast invasive carcinoma (BRCA).

As an adenosine analog, cordycepin (CD, C10H13N5O3, molecular weight 251.24) has increasingly attracted attention from both scientists and clinicians in the medical community. CD is a natural product of *Ophiocordyceps sinensis* (Berk.) or *Cordyceps militaris* Link, and its anti-cancer drug has been reported to affect tumor proliferation, invasion, and migration both *in vitro* and *in vivo* 19-22. It is worth investigating the regulatory role of ENG and the potential clinical significance of CD in BRCA or triple-negative breast cancer (TNBC), as well as in other types of cancer.

DNA methylation levels in gene loci could intricately regulate its expression. DNA methylation is a repressive mark predominantly found on CpG islands in somatic cells, suppressing genetic transcriptional activation in cell-type-specific manners, including in tumor cells 23. ENG has been identified as a tumor-suppressor gene in many cancers, including ESCC. Wong *et al.* found methylated sequences of ENG in both an ESCC cell line panel and clinical patient samples 24, while Jin *et al.* further supported that ENG promoter hypermethylation is a tissue-specific, frequent event in ESCC and shows a field defect as a potential marker for early monitoring and treatment by 5-aza-2'-deoxycytidine, which reverses methylation of ENG and reactivates its expression 25. However, ENG methylation across whole gene regions in pancancer, specifically in BRCA, and its expression regulation, diagnostic, and prognostic implications remain unknown.

In the current study, we evaluated ENG/CD105 expression, DNA methylation, immune response, and CD regulation, which could act as diagnostic, prognostic, and therapeutic markers of breast cancer (BC) including BRCA.

## Materials and Methods

### Resources and bioinformatics analysis

ENG isoform details in BRCA and expression analysis between cancer tissues and matched normal tissues were conducted by gene expression profiling interactive analysis (GEPIA2) (http://gepia2.cancer-pku.cn/#analysis). ENG expression in Chinese invasive BC tissues compared with matched normal tissues, the NCBI GEO database with accession number GSE133998 26 was performed using GEO2R. ENG protein expression was performed using UALCAN (https://ualcan.path.uab.edu/cgi-bin/ualcan-res-prot.pl), an interactive web resource for analyzing cancer OMICS data 27. DNA methylation analysis for ENG was performed by DNMIVD (http://119.3.41.228/dnmivd/query_gene/?cancer=pancancer&gene=ENG), a comprehensive annotation and interactive visualization database 28. Diagnostic and prognostic models were also analyzed by DNMIVD for BRCA in ENG using CpG islands (cg05050341, cg13458609, cg13761843, cg14055970, cg14185922, and cg24910675). Survival analysis for ENG expression in BC was conducted by Kaplan-Meier Plotter (https://kmplot.com/analysis/index.php?p=service) 29. Tumor-immune system interactions were analyzed via TISIDB (http://cis.hku.hk/TISIDB/browse.php?gene=ENG), which integrates multiple heterogeneous data types 30. The ENG protein expression was analyzed by UALCAN (The University of ALabama at Birmingham CANcer data analysis Portal) (https://ualcan.path.uab.edu/cgi-bin/CPTAC-Result.pl?genenam=ENG&ctype=Breast), and in there, the Z-value represents the standard deviation from the median across samples for this breast cancer type 31.

### Western blotting

The rabbit polyclonal antibody for ENG/CD105 was purchased from Wuhan Sanying (Cat #: 10862-1-AP, Proteintech Group, Inc, USA). β-actin was considered as an internal control. Western blotting was performed using mouse tumor tissues. A PVDF membrane was used and saturated with 5% milk in 1×TBST buffer to block free binding sites. Under constant agitation, the blocked membrane was then incubated with primary antibodies overnight at 4 °C. The antibody for ENG/CD105 was used at a 1:4000 dilutions. After washing the membrane with 1×TBST for 15 min thrice, suitable horseradish peroxidase (HRP) -coupled secondary antibodies were incubated for 1~3 hr. The membrane was washed again with 1×TBST for 15 min thrice. Lastly, the signals on the membranes were detected under the image scanner (Gene Company Limited, USA) by adding the suitable substrate 19. All experiments were repeated three times.

### Immunohistochemistry (IHC)

The CD105/ENG antibody for IHC was the same as used in western blotting and was purchased from Wuhan Sanying (Cat # 10862-1-AP, Proteintech, USA). The IHC protocols were previously described using Chinese BC samples (Patient ID:18-23900) 32, 33. For details, 5μm paraffin sections were de-paraffinized in fresh xylene for 10 min × 3 times, and hydrated with a series of concentrations in alcohol (100%, 95%, 80%, and 70%). Then, antigen retrieval was carried out and sections were washed with ddH_2_O thrice and treated with 3% H_2_O_2_ for 10 min. After being blocked with 5% bovine serum albumin (BSA) in PBS, the sections were incubated with primary antibody CD105/ENG (1:500) overnight at 4 °C, and then washed again. Next, the sections were incubated with an enzyme-labeled goat anti-mouse IgG polymer (Cat #: PV-9000, ZSGB-Bio, CN) at room temperature for 1 h. After washing in PBS, they were incubated with imidazole-DAB solution at room temperature, counterstained with hematoxylin for 1 min, de-hydrated with alcohol, cleared with xylene, and sealed with neutral gum. The slides were then observed under a microscope. We also analyzed ENG IHC data in The Human Protein Atlas (HPA) (https://www.proteinatlas.org/ENSG00000106991-ENG/pathology/breast+cancer#img) 34, 35.

### Mouse model for cordycepin (CD) treatments

CD was purchased from Chengdu, China, which was previously reported 19, 36. The 4T1 is a highly invasive mouse BC cell line or TNBC cell line. To establish a 4T1 mouse model, we first subcutaneously injected the cells into female mice. After 5 days of the injection, the tumor will reach approximately 1-3mm in size. Then, the mice will be administrated with CD. The sizes of the tumors in all groups and the whole body weights of the mice were measured periodically. At the endpoint of the study, the animals were sacrificed, and the whole cellular proteins were extracted from the tumor tissues by 1×EBC lysis buffer (20 mM Tris-HCl pH8.0, 125 mM NaCl, 2 mM EDTA, 0.5% NP-40) with protease inhibitors and conducted western blotting to measure the role of CD on ENG protein expression.

### Hematoxylin and eosin (H&E) staining

The above tumor sample sections were de-paraffinized and re-hydrated in three changes of xylene, washed in a series of decreasing concentrations of alcohol and then in tap water. The sections were stained in Harris's hematoxylin reagent, de-stained in 1% acid-alcohol (1% hydrochloric acid in 70% alcohol), and washed in running tap water. Next, the slides were agitated 3 times in ammonia water and rinsed in running tap water. The sections were then counterstained with 1% eosin and de-hydrated in 95% alcohol, in 2 changes of 100% alcohol for 30 sec each and cleared by 3 changes of xylene for 2 min each. Then, the slides were mounted with neutral gum and observed under a microscope.

### Molecular docking

The 3D structure of the ENG protein used for docking was obtained from the Uniprot database (Uniprot ID: P17813) using the AlphaFold prediction model available in the database. The 3D structure of CD was obtained from the PubChem database (PubChem ID: 6303). It was energy-minimized under the MMFF94 force field using AVOGADR 1.2.0 before docking. Molecular docking was conducted with the AutoDock Vina 1.1.2 software. Docking results were visually analyzed using the open-source version of PyMol.

## Results

### ENG expression is downregulated in most cancer types compared with corresponding normal tissues

**Figure 1:**
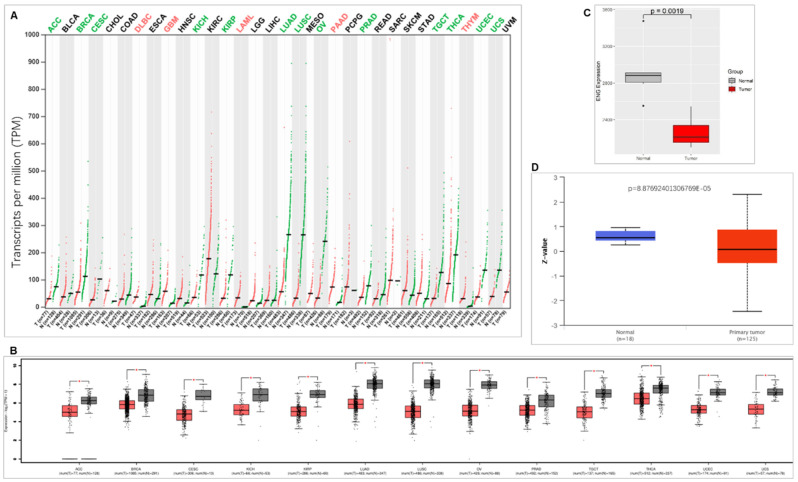
** ENG expression comparison between tumor tissues and corresponding healthy tissues.** A. Comparison of ENG expression between cancer tissues and corresponding normal tissues in different types of cancer. Red colors represent upregulated ENG expression, while green colors represent downregulated ENG expression in cancer tissues compared with corresponding healthy tissues. B. ENG expression is significantly downregulated in cancer tissues compared with corresponding healthy tissues in different types of cancer. C. ENG mRNA expression is significantly downregulated in Chinese BC tissues compared with corresponding healthy tissues. Red color indicates cancer tissues and gray color indicates matched normal tissues. D. ENG protein expression is significantly downregulated in TCGA BC tissues compared with corresponding healthy tissues. Red color indicates cancer tissues and green color indicates matched normal tissues. ENG, endoglin; TCGA, The Cancer Genome Atlas; ACC, Adrenocortical carcinoma; BLCA, Bladder Urothelial Carcinoma; BRCA, Breast invasive carcinoma; CESC, Cervical squamous cell carcinoma and endocervical adenocarcinoma; COAD, Colon adenocarcinoma; CHOL, Cholangio carcinoma; DLBC, Lymphoid Neoplasm Diffuse Large B-cell Lymphoma; ESCA, Esophageal carcinoma; GBM, Glioblastoma multiforme; HNSC, Head and Neck squamous cell carcinoma; KICH, Kidney Chromophobe; KIRP, Kidney renal papillary cell carcinoma; KIRC, Kidney renal clear cell carcinoma; LAML, Acute Myeloid Leukemia; LIHC, Liver hepatocellular carcinoma; LGG, Brain Lower Grade Glioma; LUSC, Lung squamous cell carcinoma; LUAD, Lung adenocarcinoma; MESO, Mesothelioma; OV, Ovarian serous cystadenocarcinoma; PCPG, Pheochromocytoma and Paraganglioma; PAAD, Pancreatic adenocarcinoma; PRAD, Prostate adenocarcinoma; READ, Rectum adenocarcinoma; SKCM, Skin Cutaneous Melanoma; SARC, Sarcoma; STAD, Stomach adenocarcinoma; THCA, Thyroid carcinoma; TGCT, Testicular Germ Cell Tumors; THYM, Thymoma; UCS, Uterine Carcinosarcoma; UCEC, Uterine Corpus Endometrial Carcinoma; UVM, Uveal Melanoma.

When we compared ENG expression with corresponding healthy tissues from 33 types of cancer, ENG decreases in most of the cancer types (13 types), including adrenocortical carcinoma (ACC), cervical squamous cell carcinoma and endocervical adenocarcinoma (CESC), BRCA, kidney chromophobe (KICH), kidney renal papillary cell carcinoma (KIRP), lung squamous cell carcinoma (LUSC), lung adenocarcinoma (LUAD), ovarian serous cystadenocarcinoma (OV), prostate adenocarcinoma (PRAD), testicular germ cell tumors (TGCT), thyroid carcinoma (THCA), uterine corpus endometrial carcinoma (UCEC), and uterine carcinosarcoma (UCS). Conversely, ENG expression was increased in some cancer types (5 types), including acute myeloid leukemia (LAML), lymphoid neoplasm diffuse large B-cell lymphoma (DLBC), pancreatic adenocarcinoma (PAAD), GBM, and thymoma (THYM) (Figure 1A &B). We also compared ENG expression in Chinese BC and found that ENG mRNA is significantly decreased compared with matched healthy tissues (Figure 1C). Consistently, ENG protein expression levels were also significantly downregulated in TCGA BC tissues compared with corresponding healthy tissues (Figure 1D).

**Figure 2:**
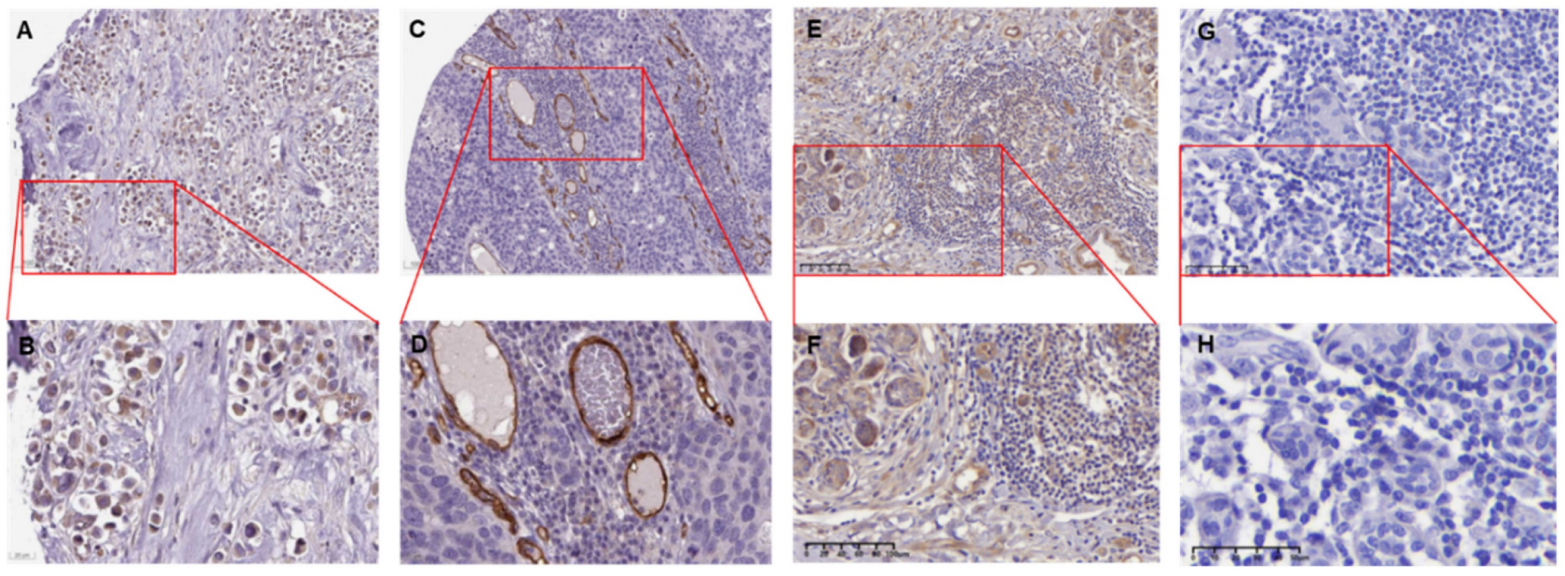
** The ENG IHC results of tumor tissues of breast cancer patients.** A. The representative IHC image shows tumor tissues from a 61-year-old female patient diagnosed with duct carcinoma breast cancer (Patient ID: 1910). The image depicts a low expression of ENG protein. [Link: https://images.proteinatlas.org/11862/158143_A_4_2.jpg]. B. An enlarged image of the highlighted area in panel (A). C. The representative IHC image shows tumors from an 83-year-old female patient diagnosed with duct carcinoma breast cancer (Patient ID: 2160). [Link: https://images.proteinatlas.org/72873/157125_A_4_6.jpg]. D. An enlarged image of the highlighted area in panel (C). E. The representative IHC image shows tumors from a 46-year-old Chinese female patient diagnosed with duct carcinoma breast cancer (BRCA) (Patient ID:18-23900). F. An enlarged image of the highlighted area in panel (E). G. The representative IHC image without ENG antibody as a negative control. H. An enlarged image of the highlighted area in panel (G). The image depicts no expression of the ENG protein in tumor cells. IHC, immunohistochemistry. ENG, endoglin.

IHC confirmed the above results in BC patients (Figure 2) and showed low or undetectable levels of ENG expression in BRCA tissues, which is located in the cytoplasmic/ membranous.

### ENG mutations across multiple types of cancer and prognosis

**Figure 3:**
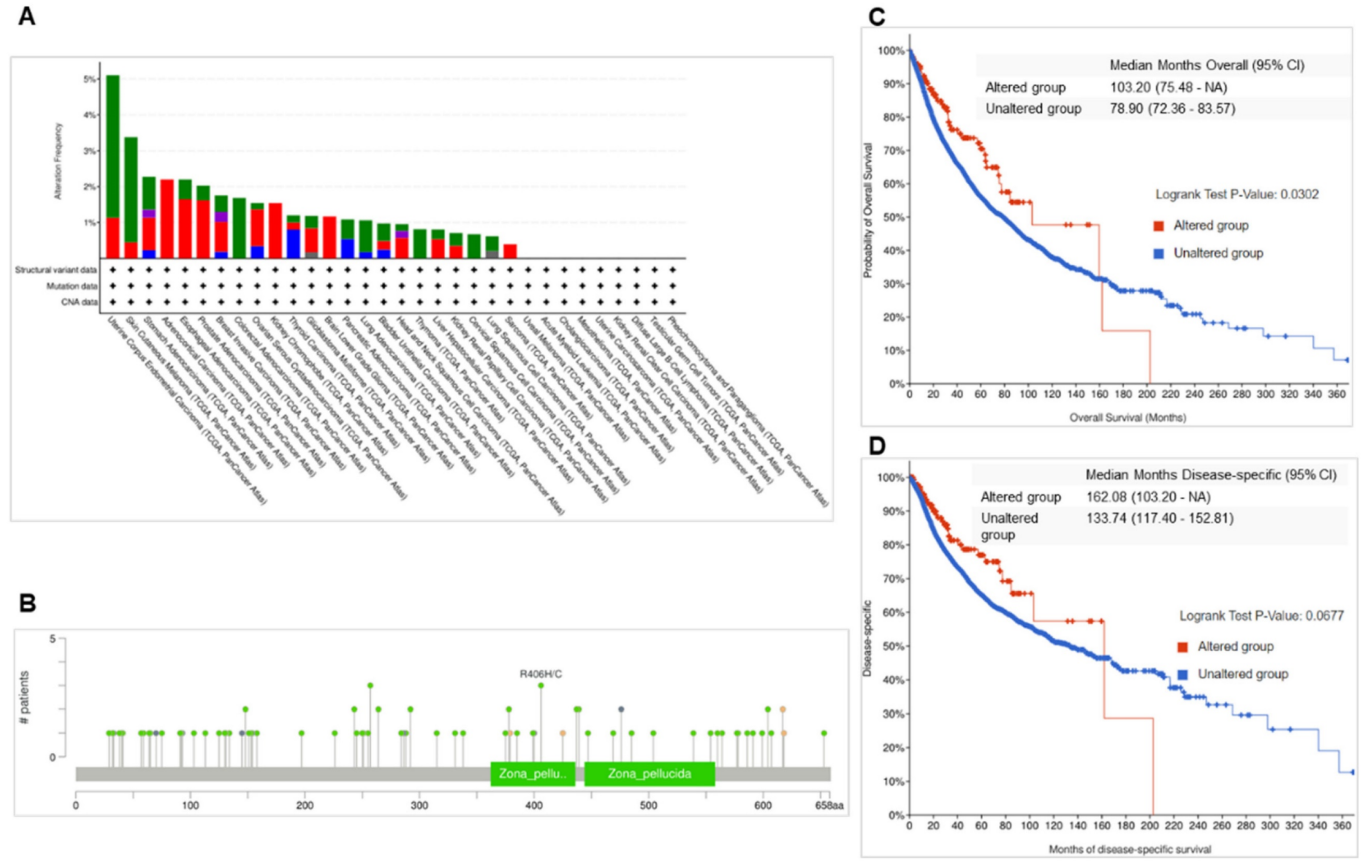
** ENG mutations in multiple types of cancer.** A. Mutation frequency of ENG in multiple types of cancer. B Mutation locations of ENG in multiple types of cancer. C. Overall survival (OS) for wild-type and mutant ENG samples. D. Disease-specific survival for wild-type and mutant ENG samples. ENG, endoglin; SV, structured variant; CNA, copy number alteration; VUS, variant of uncertain significance; TCGA, The Cancer Genome Atlas.

Mutation analysis of *ENG* across multiple types of cancer revealed that ENG DNA sequences were frequently altered in most cancer types (Figure 3A). The highest frequency of alternations was observed in UCEC, with 5.1%, including mutations at 3,97% and amplifications at 1.13% (Figure 3A). In BRCA, the *ENG* gene was altered in 1.75% of 1084 cases, including mutations in 0.46% (5 cases), structural variants in 0.28% (3 cases), amplifications in 0.83% (9 cases), and deep deletions in 0.18% (2 cases). The frequency of *ENG* alterations in BRCA ranks 7^th^ highest among the analyzed cases. These mutations are located across the entire *ENG* gene in cancer, including in the Zona pellucida-like domains (Figure 3B). Mutation types include missense, truncating, inframe, splice, and fusion mutations.

Further survival analysis for wild-type and mutant ENG cases found that overall survival (OS) for mutant ENG is significantly longer than that in the wild-type cases (Figure 3C, p=0.032, Table 1, highlighted in red). However, the survival rates for disease-specific, progression-free, and disease-free are not significantly longer than those in the wild-type cases (Figure 3D, and Data not shown).

### Correlation of ENG expression with tumor-immune systems among pan-cancer

**Figure 4:**
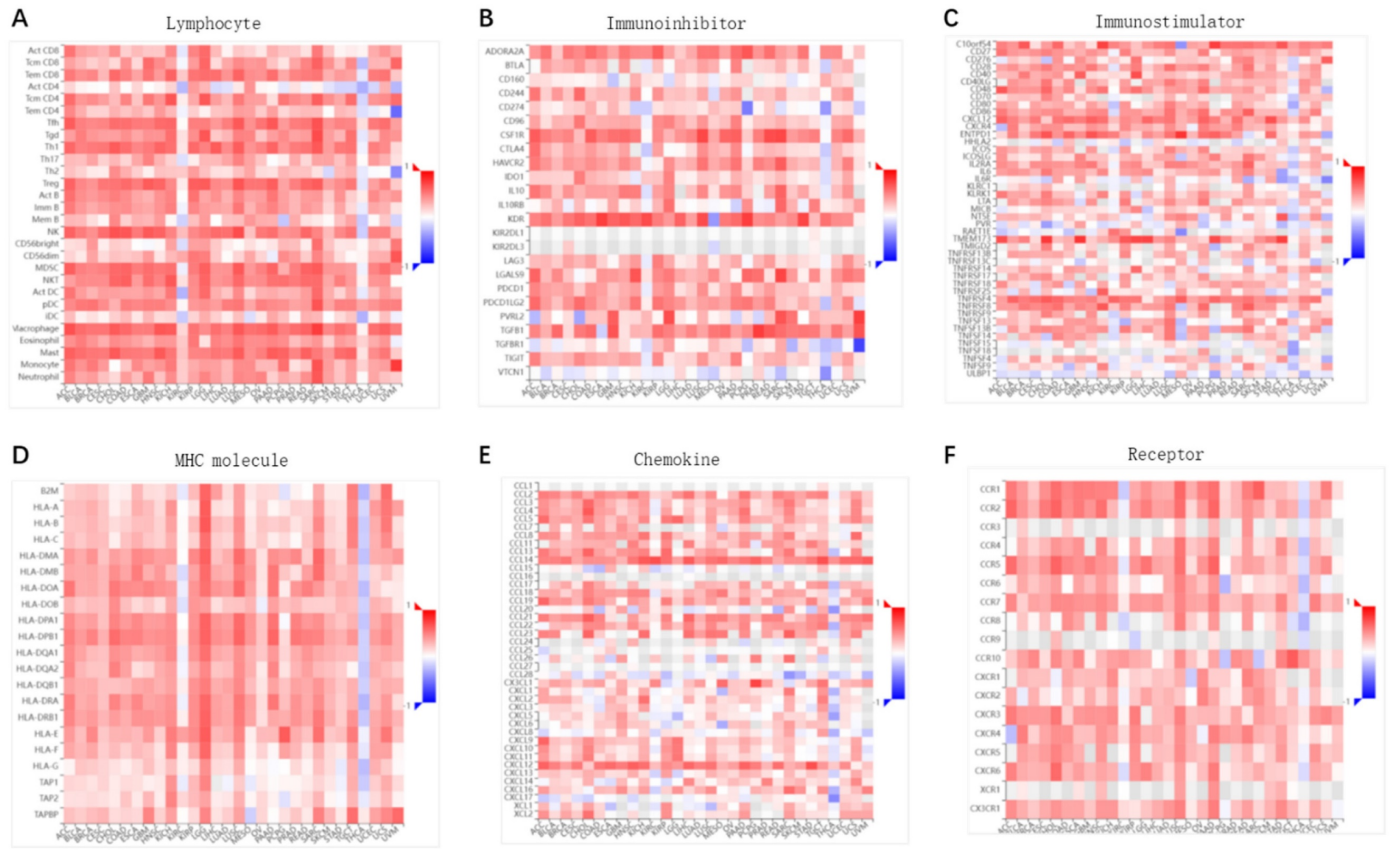
** Correlation of ENG expression with tumor-immune response/system among pan-cancer.** The correlations between ENG expression and various immune-related factors, including lymphocytes (A), immunoinhibitors (B), immunostimulators (C), MHC molecules (D), immuno-chemokine (E), and immuno-receptor (F) across pan-cancer. Y axis: human lymphocytes (A), immunoinhibitors (B), immunostimulators (C), MHCs (D), immuno-chemokine (E), or immuno-receptor (F); X-axis: cancer types. ENG, endoglin.

The indispensable role of ENG in the immune response or system may contribute to its anti-cancer capability. We conducted a correlation analysis of ENG expressions with tumor-immune systems among pan-cancer. In summary, we found a significant correlation between ENG expression and tumor-immune systems among pan-cancer (Figure 4, red colors). Specifically, it showed a positive correlation between ENG expressions and immune lymphocytes (Figure 4A), immunoinhibitors (Figure 4B), immunostimulators (Figure 4C), major histocompatibility complex (MHC) molecules (Figure 4D), immuno-chemokines (Figure 4E), or immuno-receptors (Figure 4F) across most tumor types. We assumed that ENG might be a novel target for tumor immune response and immunotherapy.

### Methylations of ENG regions in BRCA tissues and matched health tissues

**Figure 5:**
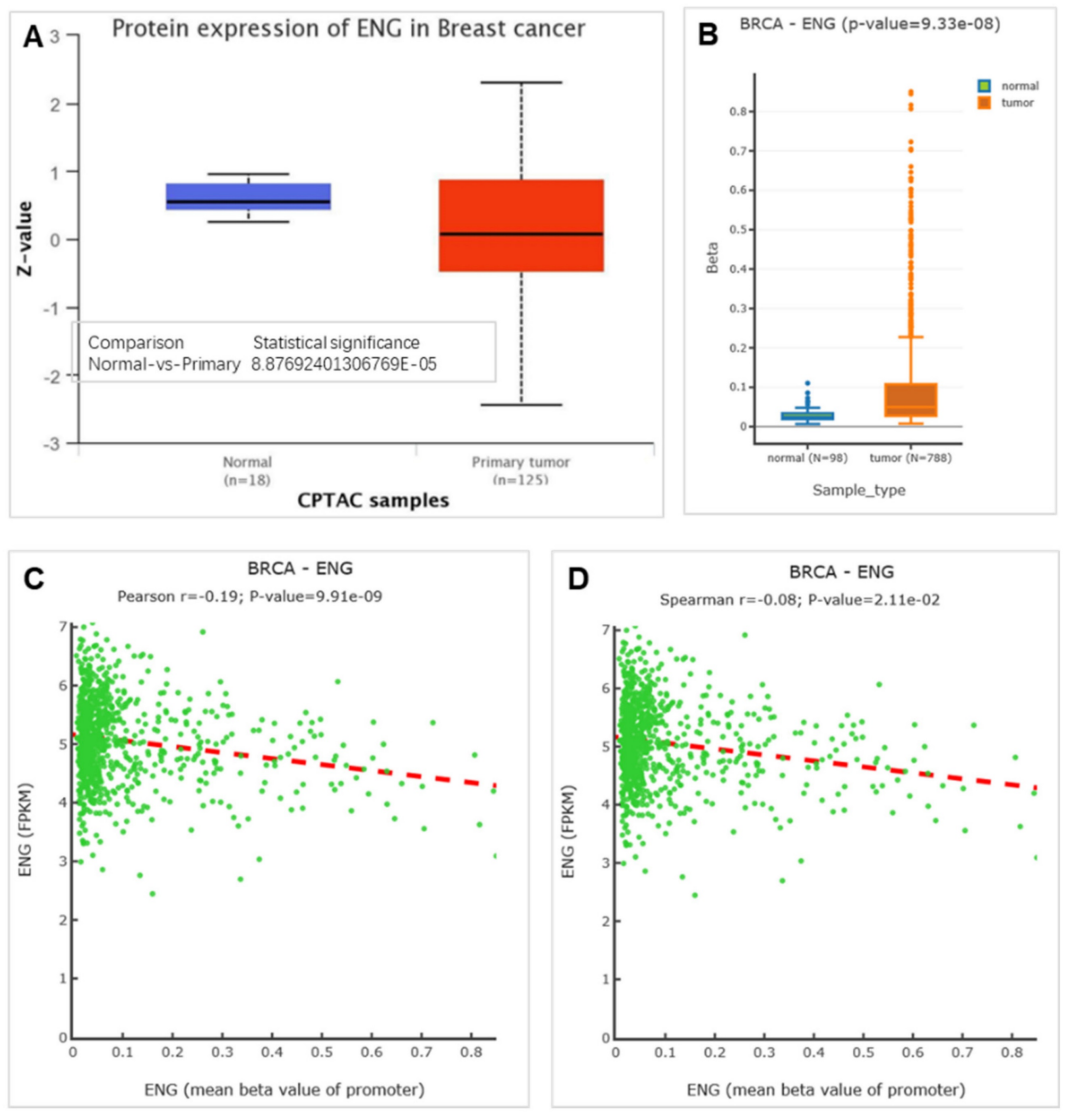
** Methylations of ENG in BRCA tissues and matched health tissues.** A. Expression for ENG protein. B. Methylations of ENG in BRCA. C &D. Pearson and Spearman correlations between ENG expression and promoter methylation respectively respectively. p< 0.05 was considered significant. ENG, endoglin; BRCA, breast invasive carcinoma.

Previous findings showed that *ENG* mRNA expression is lower in BRCA tissues compared to matched healthy tissues. We then analyzed the expression level of the ENG protein, and the results indicated that ENG protein expression is also lower in BRCA tissues compared to matched healthy tissues (Figure 5A). Further analysis of the methylation status of ENG identified six CpGs located across the entire *ENG* gene, including cg05050341, cg13458609, cg13761843, cg14055970, cg14185922, and cg24910675 (Table 2). Our results indicated that the methylation level of *ENG* is higher in BRCA tissues compared to matched healthy tissues (Figure 5B). Additionally, Pearson and Spearman correlations between ENG expression and DNA methylation revealed a significant inverse correlation (Figure 5C&D), indicating that DNA methylation might serve as a regulatory mechanism for ENG expression.

### Construction of diagnostic and prognostic models by *ENG* methylation for BRCA

**Figure 6:**
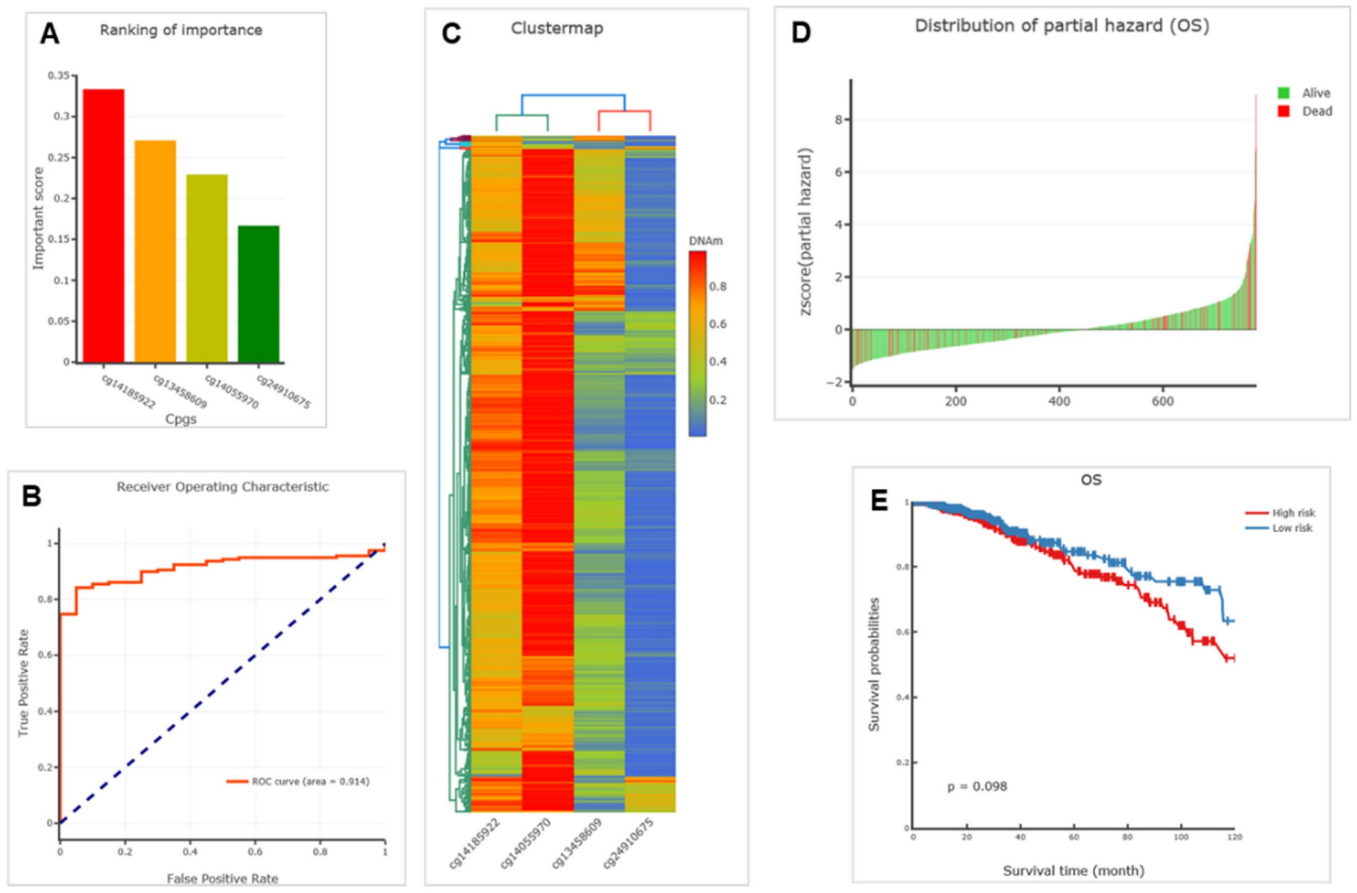
** Diagnostic and prognostic models by ENG methylation for BRCA.** A. Barplots illustrating a diagnostic model of BRCA. B. Receiver operating characteristic (ROC) curve of a logistic regression model. C. Clustering heatmap of ENG methylation profile of BRCA and matched healthy samples. D. Distribution of partial hazard for OS. E. Kaplan Meier plot of the multivariate proportional hazards regression model, categorizing patients into two groups based on high-risk or low-risk. ENG, endoglin.

By analyzing the above six CpGs, we successfully constructed diagnostic and prognostic models using *ENG* methylation for BRCA. The results are depicted in Figure 6. We revealed four CpGs (CpG sites), including cg13458609, cg14055970, cg14185922, and cg24910675 within the ENG that ranked high importance (Figure 6A). Specifically, CpG cg13458609 is located in the ENG body with an important score of 0.271, CpG cg14055970 is located in the ENG body with an important score of 0.229, cg14185922 CpG is located in the ENG body with the highest important score of 0.333, and cg24910675 is located in the 1st exon of the 5′UTR with an important score of 0.167 (Table 3). The diagnostic value assessed by the receiver operating characteristic (ROC) curve is 0.914 for the logistic regression model (Figure 6B). DNA methylation clustering heatmaps for these CpGs in BRCA are depicted in Figure 6C. Thus, these ENG DNA methylation CpGs are potential diagnostic markers that could be important in discriminating BRCA cancer from normal samples.

Further prognostic model for OS was successfully established, and we identified a significant prognostic CpG, cg14185922, using both models for univariate proportional hazards regression (endpoint=OS) and multivariate proportional hazards regression (endpoint=OS) (Table 4 &5, p= 0.007898287, highlighted in red). The distribution of partial hazard for OS associated with cg14185922 of ENG is shown in Figure 6D, while OS itself is depicted in Figure 6E, indicating that high methylation might be associated with shorter OS (high risk) in BRCA tissues compared to corresponding healthy tissues of samples (Figure 6E). Thus, *ENG* DNA methylation CpGs, specifically CpG island cg14185922, are potential prognostic markers that could be important in discriminating BRCA cancer from normal samples.

Taken together, ENG DNA high methylation CpGs, specifically CpG island cg14185922, are potential diagnostic and prognostic markers that could be important in discriminating BRCA cancer from normal samples. ENG high methylation of CpG island cg14185922 might be associated with shorter OS (high risk) in BRCA compared to matched healthy individuals.

### Isoform distribution and structure for *ENG* in BRCA

**Figure 7:**
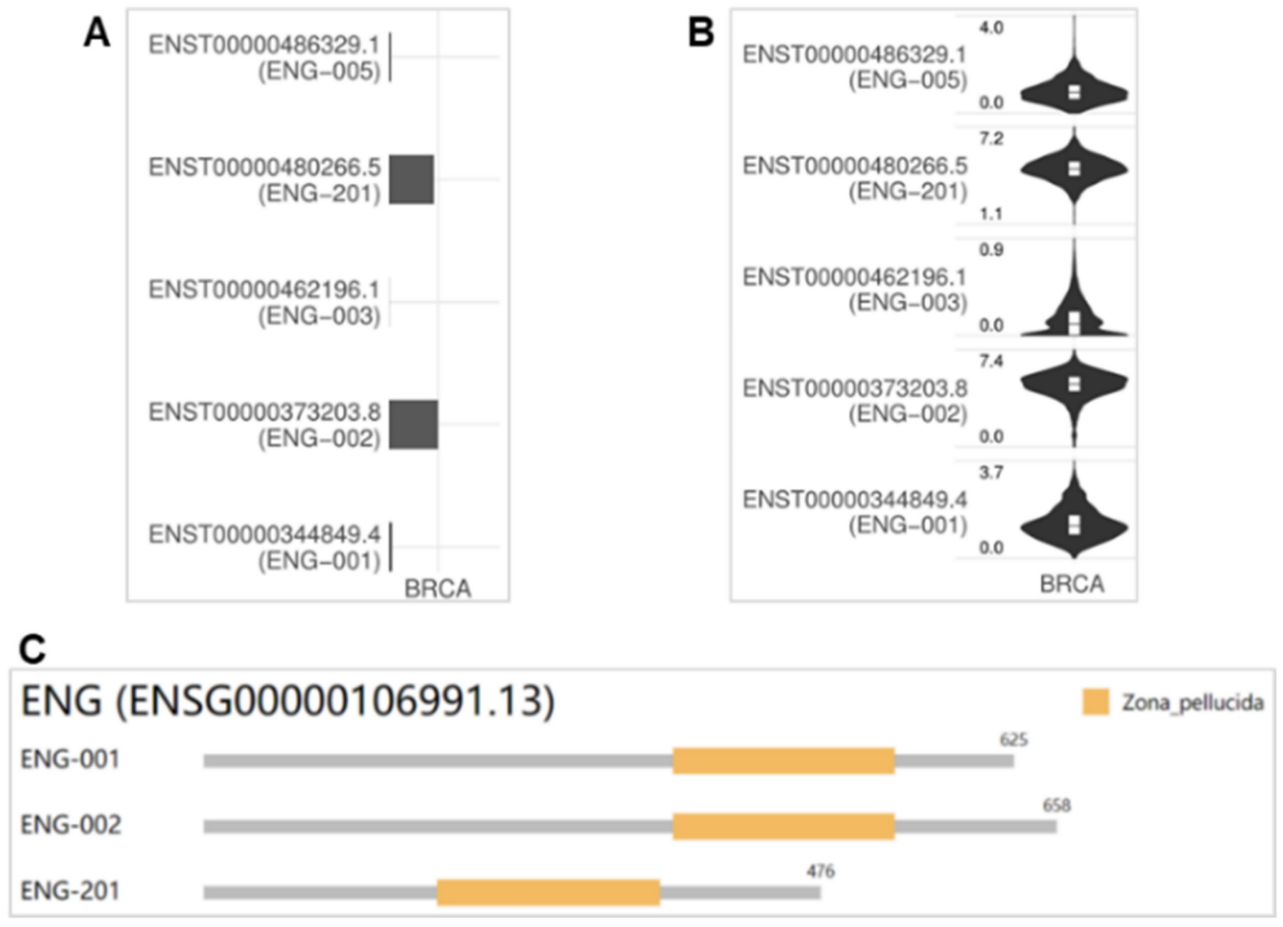
** Isoform distribution and structure for ENG in BRCA.** A &B. Bar plot and violin plot illustrating the isoform usage and the expression distribution of ENG in BRCA. C. Visualization of three isoforms and the domain structures of ENG protein in an interactive plot. ENG, endoglin.

Different isoforms with variant expressions may have different domains and roles in cancer progression. The utilizations of *ENG* isoforms ENST00000480266.5 (ENG-201) and ENST00000373203.8 (ENG-002) were high in BRCA, with ENST00000373203.8 (ENG-002) showing the highest utilization, while the other two isoforms were utilized to a very limited extent or not at all (Figure 7A). Consistently, the expression levels of isoforms ENST00000480266.5 (ENG-201) (1.1~7.2) and ENST00000373203.8 (ENG-002) (0.0~7.4) were high in BRCA [ENST00000373203.8 (ENG-002) or isoform 2 is the highest], followed by ENST00000486329.1 (ENG-005) (0.0~4.0), ENST00000344849.4 (ENG-001) (0.0~3.7), and ENST00000462196.1 (ENG-003) (0.0~0.9) (Figure 7B). The genomic structures of *ENG* isoforms in BRCA are depicted in Figure 7C. The isoforms ENG-001, ENG-002, and ENG-201 have a Zona_pellucida domain encoded by 625, 658, and 476 amino acids, respectively. Based on the expression levels and isoform usage, we conclude that the isoform ENST00000373203.8 (ENG-002) might play important roles in tumorigenesis and metastasis for BRCA.

### Relapse-free survival (RFS) analysis for BC and TNBC

**Figure 8:**
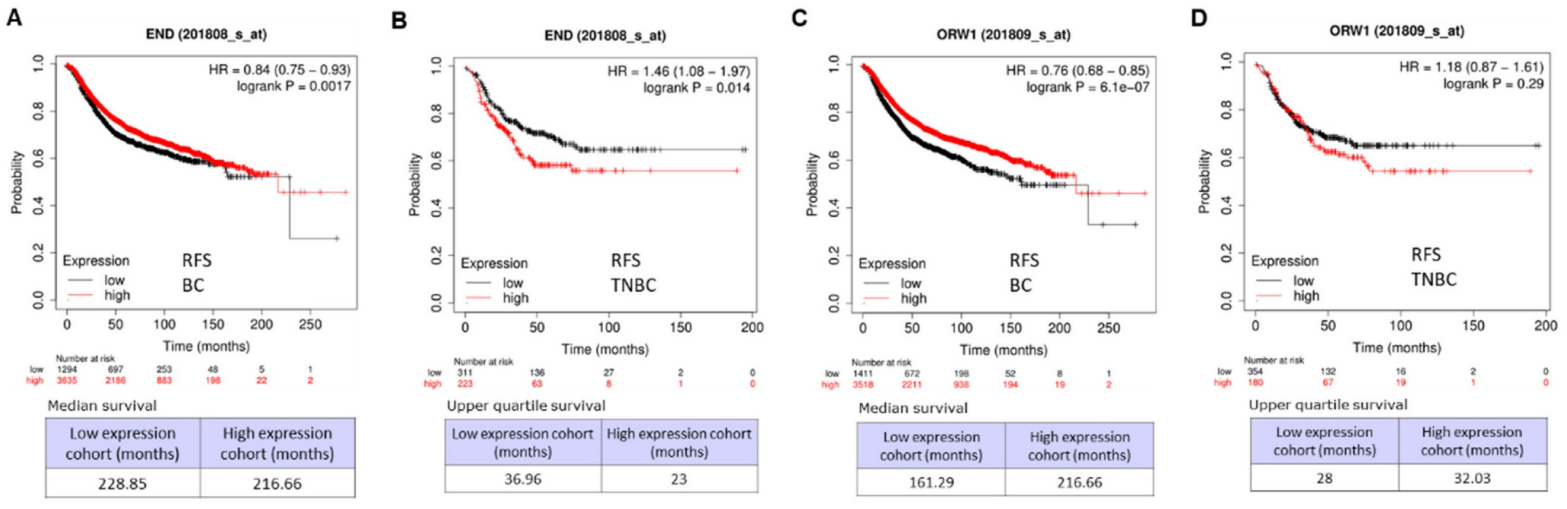
** Relapse-free survival (RFS) analysis for breast cancer.** A. RFS for breast cancer using an assay with Affy ID: 201808_s_at. B. RFS for TNBC using an assay with Affy ID: 201808_s_at. C. RFS for breast cancer using the assay with Affy ID: 201809_s_at. D. RFS for TNBC using the assay with Affy ID: 201809_s_at. TNBC, triple-negative breast cancer.

The clinical correlation between the expression of ENG and relapse-free survival (RFS) was carried out, and we revealed that high expressions of ENG significantly correlate with long RFS for BC patients in two arrays (Figure 8A &C, p < 0.001). However, in TNBC, high expressions of ENG significantly correlated with short RFS in an array Affy ID: 201808_s_at (Figure 8B, p < 0.014). In another array with Affy ID: 201808_s_at, high expressions of ENG correlated, but not remarkably, with short RFS (Figure 8D, p = 0.29). Therefore, ENG expression might be a favorable prognostic biomarker for BC patients and an unfavorable prognostic biomarker for TNBC patient survival.

### Anti-cancer agent CD upregulates ENG expression

**Figure 9:**
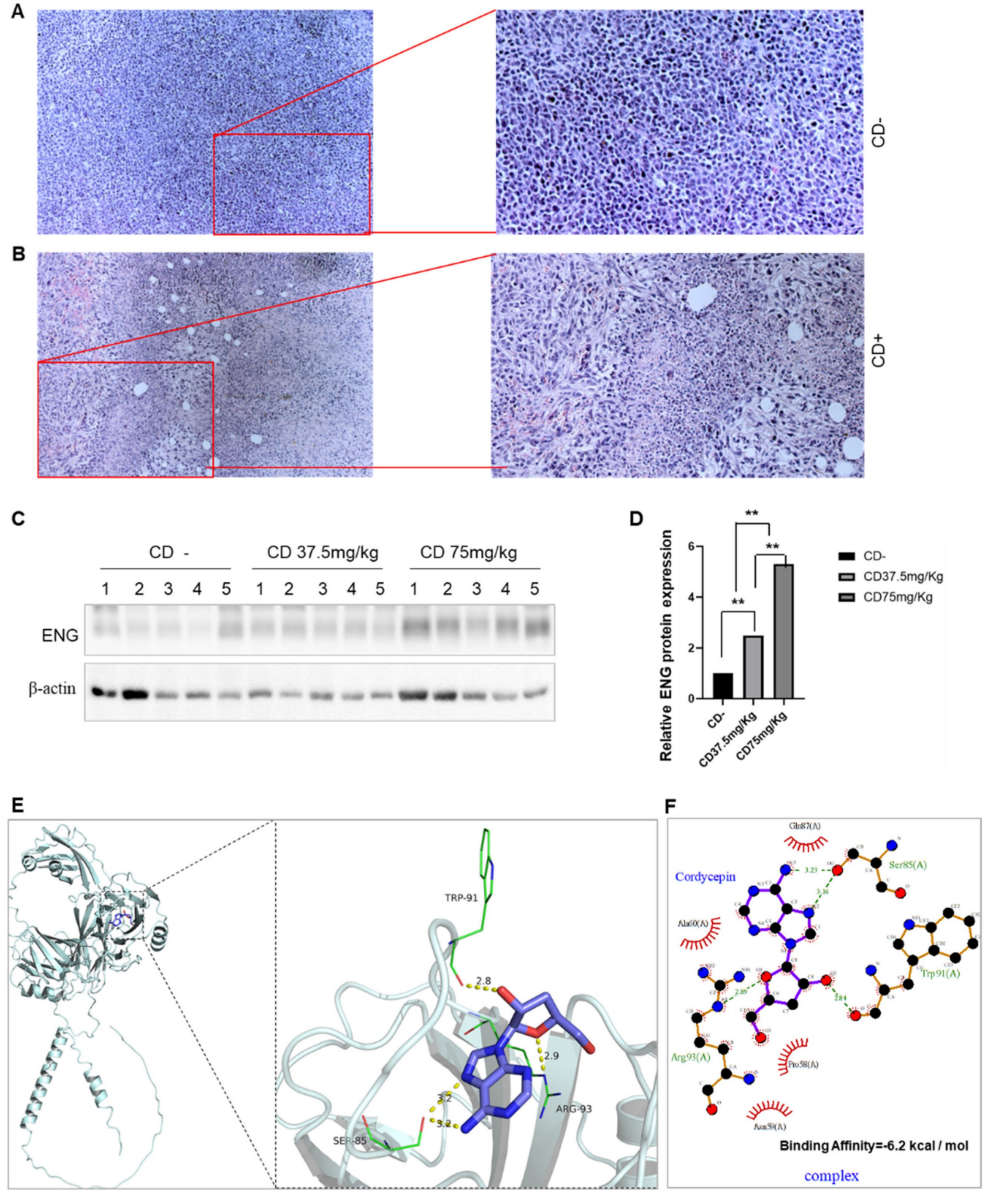
** Anti-cancer agent CD upregulates ENG expression by ENG/CD interaction.** A. Representative images for H&E staining in tumor tissues without CD treatment. B. Representative images for H&E staining in tumor tissues with CD treatment. Right panels, enlarged images from left panels. C. Western blotting results for ENG protein levels in BC tissues. D. The quantitative results from panel C of the Western blotting analysis. CD, cordycepin; ENG, endoglin; H&E staining, Hematoxylin and eosin staining. E. The ENG/CD complex based on docking for the overall binding view (left) and the local binding view (right). F. Protein and small-molecule 2D interaction map for the ENG/CD complex. The yellow dashed line represents hydrogen bonds, and green indicates the amino acids that form hydrogen bonds with the small molecule CD within the protein-binding pocket; Cartoon for the ENG protein, and purple for CD.

CD plays a role in tumor proliferation, invasion, and migration both *in vitro* and *in vivo* 19, 22. By establishing a 4T1 BC mouse model with CD treatment, CD treatment significantly decreased both tumor sizes and weights in dose-dependent manners (Data not shown). H&E staining in tumor tissues revealed that CD treatment underwent tumor cell necrosis and presented empty bubbles (Figure 9A&B). We then conducted western blotting to measure the effect of CD on ENG expression. As shown in Figure 9, the ENG protein expression is increased in dose-dependent manners upon CD treatments (Figure 9C&D). These findings suggest that CD suppresses BRCA tumor growth and metastasis, likely partially through upregulating tumor suppressor gene *ENG* expression at least.

Docking simulation technology is a convenient and effective approach to explore the interaction between small molecules and proteins. The results shown in Figure 9E&F demonstrate that, in the complex EEG/CD, CD binds to the pocket surrounding amino acids GLN 87, ALA 60 PRO 58, ASN 59, ARG 93, TRP 91, and SER 85 of the ENG protein, including forming hydrogen bonds with ARG 93, TRP 91, and SER 85 of ENG, and forming hydrophobic interactions with GLN 87, ALA 60, PRO 58, and ASN 59 of ENG. The highest binding affinity of CD to the ENG protein is -6.2 kcal / mol (Figure 9E&F). Usually values less than -5.0 kJ/mol (-1.207 kcal/mol) are considered more likely to be bound.

## Discussion

The VEGF level is correlated with the TGFβ level *in vivo* and *in vitro* 37. ENG is reported to be chiefly expressed in endothelial cells and endothelial colony-forming cells and served as a marker of angiogenesis 38, 39. The ENG protein, together with ALK1 and other endothelial receptors of the TGFβ superfamily, is necessary for vascular integrity and angiogenesis 38. ENG null mice die in utero due to impaired vasculature 40. Studies of ENG in both mice and human individuals have revealed a crucial role of the TGFβ signaling during angiogenesis and the resulting HHT1 genetic disease. Mutations of ENG caused HHT1 cause a rare genetic disorder 6. Our previous study found that the ENG/VEGFα signaling was associated with a nonsense variant of ENG causing HHT1 41. Spliced transcript variants (variant 1/2) of* ENG* have been reported to encode various isoforms, that play a role in activating monocytes, endothelial cells, and placenta, and the longer isoform (isoform 1) is predominant in normal tissues 42. Isoform 1 (GenBank number NP_001108225.1) encodes 658 amino acids with an estimated molecular weight of 70,578 Da, while isoform 2 (GenBank number NP_000109.1) encodes 625 amino acids with an estimated molecular weight of 67,542 Da. The difference between isoforms 1 and 2 is in the C-terminus of ENG. In BRCA tissues, there are five isoforms, and the utilization of ENG isoforms (ENG-201, isoform 1) and (ENG-002, isoform 2) was high; the other two were very low or absent. Consistently, the expression levels of isoforms ENST00000480266.5 (ENG-201) (1.1~7.2) and ENST00000373203.8 (ENG-002) (0.0~7.4) were high in BRCA (isoform 2 with 625 amino acids is the highest), followed by ENST00000486329.1 (ENG-005) (0.0~4.0), ENST00000344849.4 (ENG-001) (0.0~3.7), and ENST00000462196.1 (ENG-003) (0.0~0.9). The isoforms ENG-001, ENG-002, and ENG-201 have a Zona_pellucida domain encoded by 625, 658, and 476 amino acids, respectively. Based on the expression level and isoform usage, we conclude that the isoform ENST00000373203.8 (ENG-002), i.e. isoform 2, might be involved in important roles in tumorigenesis and metastasis for BRCA.

When compared with corresponding healthy tissues from 33 types of cancer tissues, ENG expression is decreased in most cancer types (13 types) and increased in some cancer types (5 types). *ENG* DNA sequences were frequently altered in most cancer types, and the OS for mutant *ENG* cases is significantly longer than in wild-type cases. The clinical correlation between the expression of ENG and RFS was further carried out in BC, and high expressions of ENG significantly correlated with long RFS. For TNBC, high expressions of ENG significantly correlated with short RFS. Therefore, ENG expression would be a favorable prognostic biomarker for BC patients and an unfavorable prognostic biomarker for TNBC.

We analyzed the *ENG* methylations and identified six CpGs. The *ENG* methylation level is higher in BRCA tissues when compared with matched health tissues. The *ENG* expression and DNA methylation revealed a significantly reverse correlation, respectively, demonstrating that DNA methylation for *ENG* may be a regulatory mechanism. By constructing diagnostic and prognostic models for *ENG* methylation in BRCA, we found four CpGs that ranked with high importance. Additionally, a prognostic model for OS was established and identified a significant prognostic CpG with both univariate and multivariate proportional hazards regression models in cg14185922. High methylation for cg14185922 of *ENG* shows shorter OS (high risk) in BRCA tissues when compared with matched health individuals. Altogether, *ENG* CpGs DNA methylations specifically for cg14185922 are potential diagnostic and prognostic biomarkers that could be important in discriminating BRCA cancer from normal samples and ENG high methylation for cg14185922 might be associated with short OS in BRCA.

Farsaci B, *et al.* 43 first reported the relation between ENG and anti-tumor immunity in murine tumor models, including BC and colon carcinoma. Their combination therapy, involving antiangiogenic tyrosine kinase inhibitors (TKIs) and therapeutic vaccines, elevated tumor-infiltrating lymphocytes (TILs), such as tumor antigen-specific CD8 T cells and upregulated the expressions of activation markers including ENG, CXCL-9, FAS-L, and CD31 in tumor-associated macrophages (TAMs) and myeloid-derived suppressor cells (MDSCs), leading to decreased tumor volumes and increased the number of tumor-free mice. Tumoral ENG was recently reported to promote immunosuppression, angiogenesis, and metastasis in renal cell carcinoma 9, 44. Nevertheless, the ENG indispensability of the immune system or response may involve its anti-cancer capability. Our analysis revealed that ENG expression is significantly correlated with tumor-immune systems among pan-cancer. Specifically, it showed a positive correlation between ENG expressions and immune lymphocytes, immuno-chemokines, immunostimulators, immunoinhibitors, MHC molecules, or immuno-receptors in most of tumor types. We assumed that ENG might be a novel target for tumor immune response and immunotherapy in pancancer including BRCA and TNBC.

CD exerts effects on tumor progression, including proliferation, invasion, and migration, both *in vitro* and *in vivo* 19-22. Our results indicated that the ENG level increases in a dose-dependent fashion upon CD treatments, suggesting that CD likely suppresses BC tumor growth and metastasis, at least partially through upregulating tumor suppressor gene *ENG* expression. Molecular docking between ENG and CD indicates their interaction may be one mechanism of CD action. Natural bioactive ingredients may block adenosine pathways for future preclinical and clinical phase studies 45.

## Conclusions

Both ENG mRNA and protein are upregulated in BRCA tissues. Five isoforms were found, and the utilization for ENG isoform (ENG-002) was the highest, which might be involved in important roles in tumorigenesis and metastasis for BRCA. *ENG* DNA was frequently altered in most cancer types, and OS for mutant *ENG* is significantly longer than in wild-type cases. High expressions of ENG remarkably correlated with long RFS for BC. *ENG* methylation level is higher in BRCA tissues and revealed a significantly reverse correlation with *ENG* expression, demonstrating that *ENG* methylation may be a regulatory mechanism. *ENG* CpGs including cg14185922 DNA methylation are potential diagnostic and prognostic biomarkers of BRCA. ENG is likely to be a novel target for tumor immune response and immunotherapy, and CD likely suppresses BC growth and metastasis at least partially through upregulating tumor suppressor gene ENG expression. Thus, our study successfully evaluated ENG/CD105 expression, DNA methylation, immune response, and CD regulation that act as a diagnostic, prognostic, and therapeutic marker of BC including BRCA. Our research also fills critical knowledge gaps in the cancer field and highlights its potential importance for the diagnosis, prognosis, and treatment of BRCA.

## Figures and Tables

**Table 1:** The survival data on pancancer patients from ENG mutation.

Survival type	Number of patients	p-value	q-value
Overall	10803	0.0302	0.121
Disease-specific	10258	0.0677	0.135
Progression Free	10613	0.477	0.636
Disease Free	5383	0.901	0.901

**Table 2:** Methylated CpGs located in ENG.

Gene	CpG	Group	Relation to island
ENG	cg05050341	TSS1500	S_Shore
ENG	cg13458609	Body	OpenSea
ENG	cg13761843	Body	OpenSea
ENG	cg14055970	Body	OpenSea
ENG	cg14185922	Body	N_Shore
ENG	cg24910675	1stExon;5'UTR	S_Shore

**Table 3:** Diagnosis model of features importance score for BRCA.

CpG	Gene	Group	Relation to island	Methylation status	Score
cg05050341	ENG	TSS1500	S_Shore	Not Sig	0
cg13458609	ENG	Body	OpenSea	Not Sig	0.271
cg13761843	ENG	Body	OpenSea	Not Sig	0
cg14055970	ENG	Body	OpenSea	Not Sig	0.229
cg14185922	ENG	Body	N_Shore	Not Sig	0.333
cg24910675	ENG	1stExon; 5'UTR	S_Shore	Not Sig	0.167

Note: This model is calculated by the xgboost algorithm, and features with importance = 0 were removed. A 2 kb sequence along up- and downstream of CpGs are called the northern (N_shore) and southern shore (S_shore) respectively. OpenSea refers to the rest of the genome (not in the region of shores or shelves).

**Table 4:** Univariate proportional hazards regression model (endpoint=OS) for BRCA.

CpG	coef	exp(coef)	se(coef)	z	p	-log2(p)	lower 95%	upper 95%
cg13458609	-0	0.85	0.461	-0.35	0.728	0.46	-1.06	0.743
cg14055970	1	3.38	1.14	1.069	0.285	1.81	-1.01	3.452
cg14185922	-2	0.13	0.781	-2.66	0.008	6.98	-3.61	-0.544
cg24910675	-1	0.5	0.871	-0.8	0.425	1.24	-2.4	1.012

**Table 5:** Multivariate proportional hazards regression model (endpoint=OS) for cg14185922 for BRCA.

CpG	coef	exp(coef)	se(coef)	z	p	-log2(p)	lower 95%	upper 95%
cg14185922	-2	0.13	0.781	-2.66	0.008	6.984	-3.606	-0.5441
